# A potential protective role of the nuclear receptor-related factor 1 (Nurr1) in multiple sclerosis motor cortex: a neuropathological study

**DOI:** 10.1093/braincomms/fcad072

**Published:** 2023-03-17

**Authors:** Jonathan Pansieri, Marco Pisa, Richard L Yates, Margaret M Esiri, Gabriele C DeLuca

**Affiliations:** Nuffield Department of Clinical Neurosciences, University of Oxford, John Radcliffe Hospital, Oxford, UK; Nuffield Department of Clinical Neurosciences, University of Oxford, John Radcliffe Hospital, Oxford, UK; Nuffield Department of Clinical Neurosciences, University of Oxford, John Radcliffe Hospital, Oxford, UK; Nuffield Department of Clinical Neurosciences, University of Oxford, John Radcliffe Hospital, Oxford, UK; Nuffield Department of Clinical Neurosciences, University of Oxford, John Radcliffe Hospital, Oxford, UK

**Keywords:** multiple sclerosis, Nurr1, neuronal loss, lymphocyte inflammation

## Abstract

Cerebral cortical inflammation and neurodegeneration are hallmark pathological features of multiple sclerosis that contribute to irreversible neurological disability. While the reason for nerve cell death is unknown, the pathogenic inflammatory role of infiltrating lymphocytes is likely an important contributor. The nuclear receptor-related factor 1 counteracts inflammation in animal models of multiple sclerosis, and protects against neuronal loss in other neurodegenerative disorders, but its expression in post-mortem multiple sclerosis tissue is not known. This study aims to investigate the nuclear receptor-related factor 1 expression in multiple sclerosis motor cortex and evaluate its relationship with motor cortical pathology. To accomplish this, an autopsy cohort of pathologically confirmed multiple sclerosis (*n* = 46), and control (*n* = 11) cases was used, where the nuclear receptor-related factor 1 expression was related to neuronal and lymphocytic densities. Motor cortical nuclear receptor-related factor 1 was overexpressed in multiple sclerosis compared to control cases. Increased nuclear receptor-related factor 1 expression positively associated with neuronal densities, especially when present in nucleus of neurons, and associated with decreased CD8+ cytotoxic lymphocyte density. Our findings expand the current knowledge on nuclear receptor-related factor 1 in neurological diseases, and support the hypothesis that nuclear receptor-related factor 1 may play a dual neuroprotective role in multiple sclerosis by influencing inflammatory and neurodegenerative processes. Future studies elucidating the influence of nuclear receptor-related factor 1 on these processes in multiple sclerosis may cast light onto novel targets that may be modulated to alter clinical outcome.

## Teaser

Inflammation/neurodegeneration is a pathological hallmark of multiple sclerosis. Pansieri *et al*. report that nuclear receptor-related factor 1 upregulation in multiple sclerosis cortex associates with neuronal density and lymphocytic inflammation. Nuclear receptor-related factor 1 may play a dual neuroprotective role; elucidating its influence in multiple sclerosis may highlight targets to alter clinical outcome.

## Graphical Abstract

**Graphical Abstract:**
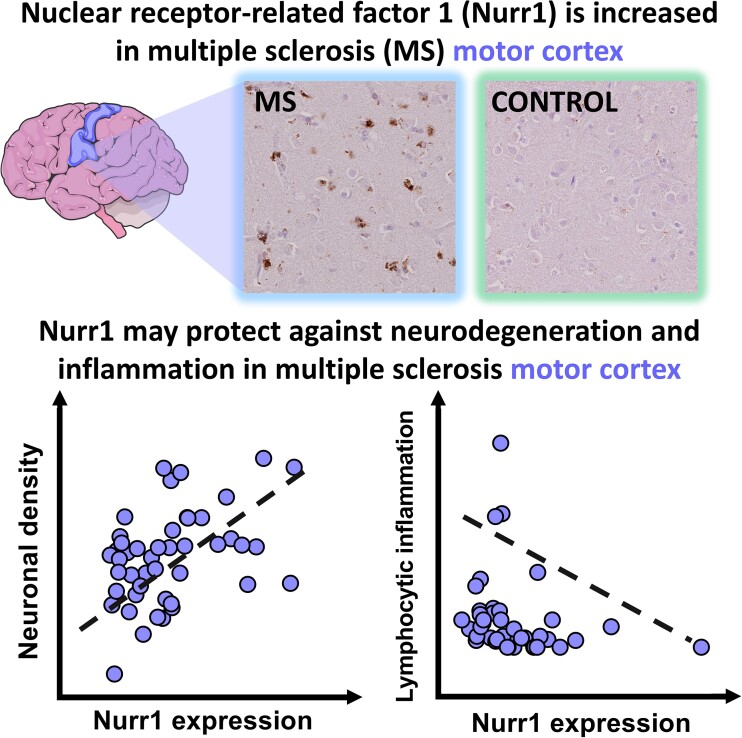

## Introduction

Multiple sclerosis is a chronic immune-mediated neurological disease characterised by inflammation, demyelination and neurodegeneration that results in disability and reduction of quality of life.^1^ Motor dysfunction is a characteristic feature of the progressive phase. Pathological studies suggest that neuronal loss in the motor cortex contributes to this irreversible motor decline in multiple sclerosis.^2^ Several lines of evidence also implicate T-lymphocytes in multiple sclerosis-related immune dysfunction that contributes to neurodegeneration.^3^ However, the causes of and relationship between inflammation and neuronal loss remain poorly understood, highlighted by the marginal impact of current treatments on long-term clinical outcome.^4^

There is an urgent need to find druggable targets to prevent the deleterious inflammatory and neurodegenerative processes in multiple sclerosis. The nuclear receptor-related factor 1 protein, termed Nurr1, is a transcription factor which may represent a promising target, with the ability to modulate expression of inflammatory factors,^5,6^ while maintaining neuronal survival in other neurodegenerative disorders.^7,8^ Recent studies have shown a protective role of Nurr1 in experimental autoimmune encephalomyelitis, an animal model of multiple sclerosis, by using Nurr1 knock-down models, which have also been shown to have more aggressive experimental autoimmune encephalomyelitis related to increased inflammation in the spinal cord.^9^ Blood samples from people with multiple sclerosis show impaired Nurr1 expression in T-cells,^10^ which exacerbates pro-inflammatory mediator release, and alters neuronal structure leading to myelin damage. Further, Nurr1 is a transcription factor that can activate or repress a wide range of genes. Cell culture models have shown that nuclear translocation of Nurr1 is influenced by specific nuclear import and export signals linked to the inflammatory milieu.^11^ Thus, the multifaceted Nurr1 protein can impact inflammatory and neurodegenerative mechanisms, and, therefore, is an attractive candidate to examine in central nervous system diseases where these processes feature. The expression of Nurr1 and its relationships to inflammation and neurodegeneration in the multiple sclerosis brain is not known and is the focus of the current study.

We provide first evidence that Nurr1 is upregulated in the multiple sclerosis motor cortex compared to controls, and is associated with reduced neurodegeneration and inflammation. Multiple sclerosis cases with abundant Nurr1 expression exhibit decreased neuronal loss and parenchymal CD8+ inflammation compared to multiple sclerosis cases with low Nurr1 expression levels. Together, these findings suggest that Nurr1 counteracts both neurodegeneration and inflammation processes in progressive multiple sclerosis, making it a promising target to halt disability accumulation characteristic of this disease.

## Material and methods

### Study population

A post-mortem cohort of pathologically confirmed multiple sclerosis cases (*n* = 46) and non-neurological controls (*n* = 11) from the UK MS Tissue Bank was used (Table 1). All autopsy material was obtained with the relevant ethics committee approval (REC 08/MRE09/31+5) according to the regulations outlined in the UK Human Tissue Act (2004). For each case, brain weight was recorded before sampling.

**Table 1:** Demographics of multiple sclerosis and control cohort

	Multiple sclerosis cases (*n* = 46)	Controls (*n* = 11)
Age of death (y)	63 (range 40–92)	76 (range 68–91)
Duration of disease (y)	31 (range 11–58)	n/a
Sex	M = 12 F = 34	M = 7 F = 4
Clinical course	Primary progressive multiple sclerosis = 4; secondary progressive multiple sclerosis = 37; relapsing–remitting multiple sclerosis = 1; unknown = 4	n/a
Brain weight (g)	1159 (range 894–1380)	1330 (range 1072–1628)
Time to wheelchair (y)	19 (range 3–50)	n/a
PM interval (h)	18 (range 7–38)	28 (range 10–52)
Multiple sclerosis cohort at extremes of supragranular Nurr1 expression (GAD+ neurons and CD8+ inflammation assessment)		
	**Multiple sclerosis: low Nurr1 (*n* = 10)**	**Multiple sclerosis: high Nurr1 (*n* = 10)**
Age of death (y)	63 (range 46–77)	63 (range 43–80)
Duration of disease (y)	27 (range 19–39)	31 (range 11–58)
Sex	M = 3 F = 7	M = 3 F = 7
Clinical course	Primary progressive multiple sclerosis = 1; secondary progressive multiple sclerosis = 9	Primary progressive multiple sclerosis = 1; secondary progressive multiple sclerosis = 9
Brain weight (g)	1209 (range 1050–1380)	1151 (range 1045–1291)
Time to wheelchair (y)	20,5 (range 6–35)	19,6 (range 4–50)
PM interval (h)	17,7 (range 10–27)	22,5 (range 13–28)

n/a = not applicable; M = male; F = female; PM = post-mortem. Data on time to wheelchair was only available in 38 of the 46 cases studied.

### Quantitative neuropathology: immunohistochemistry (IHC) on multiple sclerosis human post-mortem tissues

Briefly, adjacent sections (6 μm thick) of formalin-fixed paraffin-embedded (FFPE) motor cortical tissue were deparaffinised, and conventional antigen retrieval procedures were applied, as listed in Table 2. FFPE sections were labelled with primary antibodies for Nurr1 (Abcam #ab41917), lymphocytes (CD8, Dako #IS623; CD3, Dako #A0452) and neurons (NeuN, Abcam #177487; GAD65/67, Milipore #AB1511) revealed by 3,3′-diaminobenzidine (DAB) staining after secondary antibody incubation labelled with horseradish peroxidase (Dako REAL EnVision Detection System, #K5007), and counterstained using haematoxylin, as previously described.^12^ To minimise non-specific background staining, we used a commercial protein blocker (Protein Bloc Serum-Free, Dako #X0909) prior to application of the primary antibody. The omission of primary and secondary antibodies separately was used as negative controls (Supplementary Fig. 1).

**Table 2:** Antibodies used in staining procedures presented in this article

Target	Primary antibody	Antibody dilution	Clone	Antigen retrieval	Incubation settings
PLP	Biorad #MCA839G	1:1000	Monoclonal	Citrate pH6 microwave	1 h RT
CD8	Dako #IS623	1:5	Monoclonal	Citrate pH6 autoclave	1 h RT
CD3	Dako #A0452	1:100	Polyclonal	Tris-EDTA pH 9 autoclave	1 h RT
Nurr1	Abcam #ab41917	1:1500	Monoclonal	Tris-EDTA pH 9 autoclave	ON 4°C
Nurr1	Santacruz #Sc376984	1:50 (IF)	Monoclonal	Tris-EDTA pH 9 autoclave	ON 4°C
NeuN	Millipore #MAB377	1:400	Monoclonal	Citrate pH6 autoclave	1 h RT
NeuN	Abcam #ab177487	1:200 (IF)	Monoclonal	Tris-EDTA pH 9 autoclave	ON 4°C
GAD	Millipore #ab1511	1:200	Polyclonal	Citrate pH6 autoclave	1 h RT
TMEM119	Sigma #HPA051870	1:200 (IF)	Monoclonal	Tris-EDTA pH 9 autoclave	ON 4°C
GFAP	Abcam #ab4674	1:1000 (IF)	Monoclonal	Tris-EDTA pH 9 autoclave	ON 4°C

IF = immune-fluorescence.

Motor cortical demyelination was defined by complete loss of myelin using PLP immunostaining, as previously published and illustrated in Supplementary Fig. 2.^13^ Due to tissue availability, only seven controls were immunostained for NeuN, CD3 and CD8 markers.

To ensure Nurr1 specific cytoplasmic expression, immunohistochemistry for Nurr1 (Abcam #ab41917) was performed using the alkaline phosphatase substrate system (Vector Laboratories, #SK5100) with biotinylated secondary antibodies and avidin/biotynilated enzyme complex (Vector Laboratories, #AK5200) (Supplementary Fig. 1) per protocol. In parallel, to distinguish the Nurr1 expression pattern from lipofuscin, we used Sudan black B to label lipofuscin, as previously published.^14^ Each section was counterstained with haematoxylin.

Fluorescent double-labelling using the same primary antibodies for Nurr1 with neuronal (NeuN) labelling was performed in a subset of multiple sclerosis cases (*n* = 3) and controls (*n* = 3) using a well-established protocol and relevant secondary antibodies [i.e. Alexa Fluor 594 (anti-mouse, ThermoFisher, # A-21203) and Alexa Fluor 647 (anti-rabbit, ThermoFisher, # A32795), respectively]. To minimise autofluorescence, we used a commercial quencher designed specifically against intrinsic fluorescence of lipofuscin (TrueBlack Lipofuscin Autofluorescence Quencher, Biotium #23007).

### Investigation of motor cortical Nurr1 expression, neuronal and lymphocyte densities

All markers have been quantified in the parenchyma of multiple sclerosis cases and controls within pre-defined spaced trajectories perpendicular to the subpial surface of the motor cortex, as previously described.^13^ For each trajectory, two fields of view (FOVs) of the same size were applied to motor cortical grey matter (GM) layers, with the exception of layer 3 in which four FOVs were used due to the larger size of this layer. Cortical layers 1–3 were defined as supragranular cortex, and cortical layers 4–6 were defined as infragranular cortex. Using this established method, ∼3000 FOVs were quantified for each marker in our cohort (>15 000 FOVs in total). Each case was scanned using Aperio ScanScope AT Turbo digital scanner at 40 × resolution.

Nurr1 expression was quantified in each FOV by an established semi-automatic colour-based extraction method using Qupath software, and expressed as chromogen positive pixels/mm^2^.

Neuronal (NeuN+, GAD+) and lymphocytic (CD3+, CD8+) densities were quantified in each FOV by manually counting the number of positive cells, based on chromogen positivity and morphological features, expressed as cells/mm^2^, as previously described.^12^ As an example, >80 000 NeuN+ neurons were counted. To further evaluate the relationship between neuronal density and Nurr1 expression within the multiple sclerosis cohort, multiple sclerosis cases were divided into two subgroups, defined by the median value of Nurr1 expression in supragranular layers where Nurr1 expression shows meaningful variation (N.B. infragranular cortical layers, particularly layer 5, demonstrate similarly high expression of Nurr1 in both multiple sclerosis and controls). Scores equal to the median value were removed resulting in *n* = 22 multiple sclerosis cases in each of the high and low Nurr1 expression groups.

On close inspection of Nurr1 expression in pyramidal neurons, sub-cellular localisation differences were observed (i.e. cytoplasmic and nuclear) which were not captured by our chromogen pixel counting method. Therefore, a separate semi-quantitative score was devised to assess nuclear and cytoplasmic Nurr1 expression in neurons located in cortical projection layers 3 and 5 given their functional significance in motor function. In each FOV, cytoplasmic and nuclear Nurr1 expression was assessed as follows: 0 (mostly nuclear, i.e. >50% of Nurr1 is nuclear in selected FOVs), 1 (similar cytoplasmic-nuclear expression) or 2 (mostly cytoplasmic, i.e. >50% of Nurr1 is cytoplasmic in selected FOVs). We further compared the nuclear or cytoplasmic expression pattern of Nurr1 in multiple sclerosis cases segregated by high and low Nurr1 expression in each of cortical layer 3 and layer 5, separately, by dividing the multiple sclerosis cases into two subgroups above and below median score of Nurr1 subcellular localisation. Scores equal to the median value were removed (three cases in layer 3). To facilitate the understanding of the subheadings, these subgroups are labelled ‘nuclear’ Nurr1 below the median value, and ‘cytoplasmic’ Nurr1 above the median value.

Cases lying at the extremes of Nurr1 expression (*n* = 10 with high Nurr1 expression and *n* = 10 with low Nurr1 expression) matched for age, sex, clinical course, disease duration and brain weight (Table 1) were selected for evaluation of GAD+ neurons and lymphocyte density.

### Statistical analyses

Relationships between Nurr1 expression and neuronal/lymphocyte counts were explored. Where data were not normally distributed, Mann-Whitney test was used to assess quantitative differences between multiple sclerosis cases and controls, and within the multiple sclerosis cohort when comparing subgroups. Correlations were performed using Spearman rank-correlation coefficients. Data are presented with ± mean deviation of the mean. Two-tailed tests were used, where *P* < 0.05 were considered significant. Statistical tests were performed on GraphPad Prism software (version 8.4.2).

In order to test the combinatory effect of Nurr1 expression and neuronal subcellular localisation on neuronal densities within the multiple sclerosis cohort, we applied a generalised linear model using NeuN+ counts in the supragranular layer as dependent variable and Nurr1 subcellular localisation in layer 3 neurons, Nurr1 expression in the supragranular layers and their interaction term as independent variables. In the manuscript, we report the model fit as likelihood ratio chi square, and the effect of each independent variable as Wald chi square, together with their *P*-values. Sensitivity analyses included (i) the addition of sex, age and post-mortem interval to the model and (ii) use of layer 3 specific NeuN counts and Nurr1 expression values in the model instead of supragranular values. The same analyses were applied to layer 5 but were not statistically significant.

## Results

### Demographic features of multiple sclerosis cohort

Clinical details of the cohort can be found in Table 1. The majority of multiple sclerosis cases were female (female, 34 of 46 = 74%, male, 12 of 46 = 26%) and the median age was 63 years. Most multiple sclerosis cases were classified as secondary progressive multiple sclerosis (37 of 46 = 80.4%), followed by four primary progressive multiple sclerosis (8.6%), one relapsing–remitting multiple sclerosis (2.2%) and four cases with unknown clinical course (8.7%).

### Nurr1 expression is increased, and its subcellular localisation in neurons differs in multiple sclerosis motor cortex compared with controls

**Figure 1:**
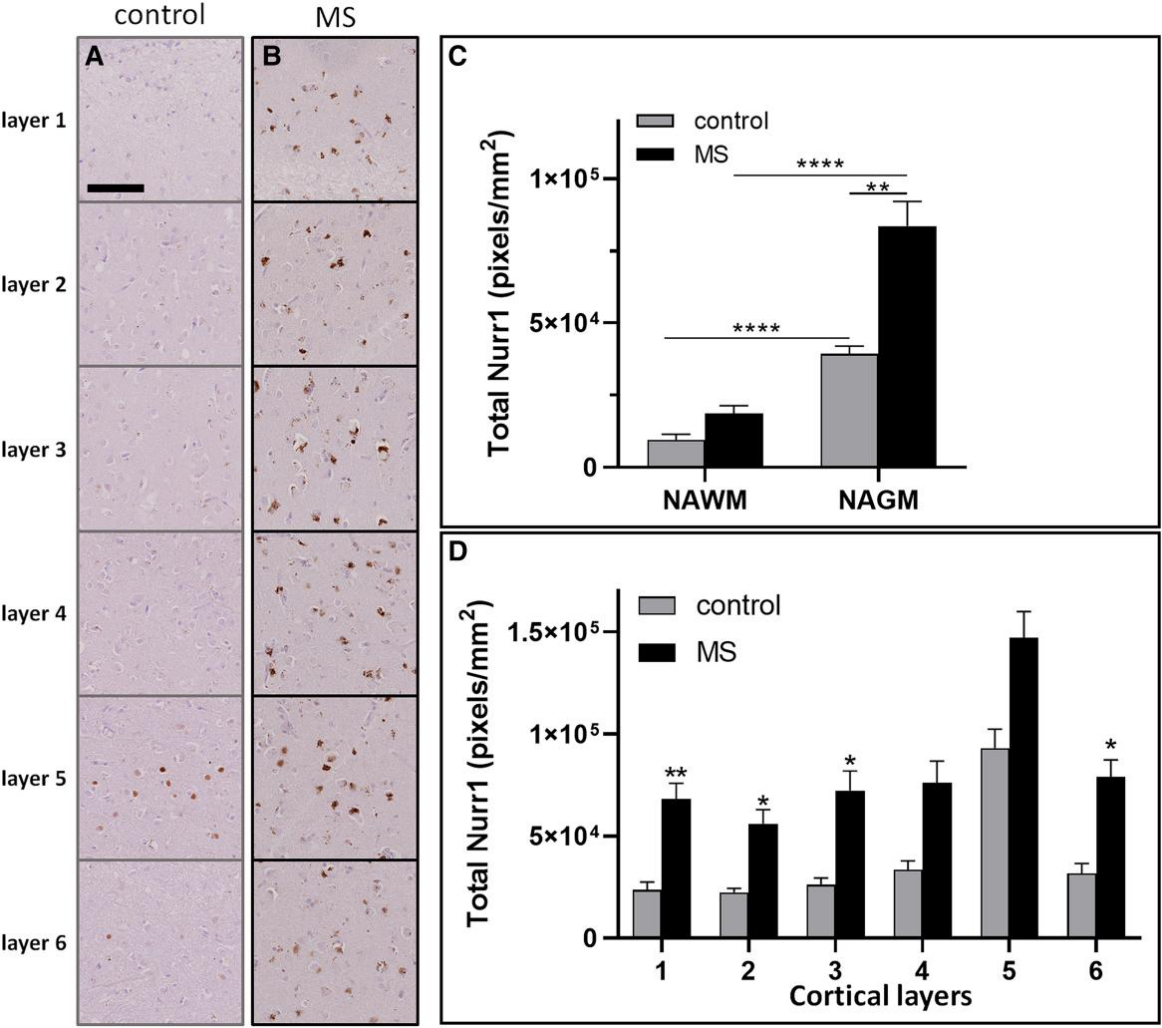
**Nurr1 expression in multiple sclerosis and control motor cortex.** Nurr1 expression in the six cortical layers of (**A**) control and (**B**) multiple sclerosis cases revealed by DAB immunostaining. Quantitation of Nurr1 expression using a chromogen extraction method, comparing (**C**) normal-appearing white matter and normal-appearing grey matter and (**D**) each cortical layer, in control and multiple sclerosis cases. Mann-Whitney tests, data presented as mean ± SEM. *P < 0.05; **P < 0.01; ****P < 0.0001; scale bar 100 μm.

The extent of Nurr1 expression was compared between multiple sclerosis and control cases (Fig. 1A and B).

Increased Nurr1 expression was seen in normal-appearing grey matter (NAGM) compared with normal-appearing white matter (NAWM) in both multiple sclerosis and control cases (Fig. 1C). Relative to controls, Nurr1 expression was increased in the GM of multiple sclerosis motor cortex (multiple sclerosis, 82 755 ± 45 735 pixels/mm^2^ versus control, 38 738 ± 6977 pixels/mm^2^; *P* = 0.002; Fig. 1C). Layer-by-layer quantification showed that Nurr1 expression was more abundant in the functionally-relevant GM layer 5 compared to other cortical layers in both multiple sclerosis and control cases (Fig. 1D). Nurr1 expression was increased in multiple sclerosis compared to control cases in each supragranular layer (layer 1, *P* = 0.0098; layer 2, *P* = 0.013; layer 3, *P* = 0.028). In contrast, in infragranular layers, Nurr1 expression was increased only in layer 6 (layer 4, *P* = 0.070; layer 5, *P* = 0.080; layer 6, *P* = 0.017, Fig. 1D).

**Figure 2:**
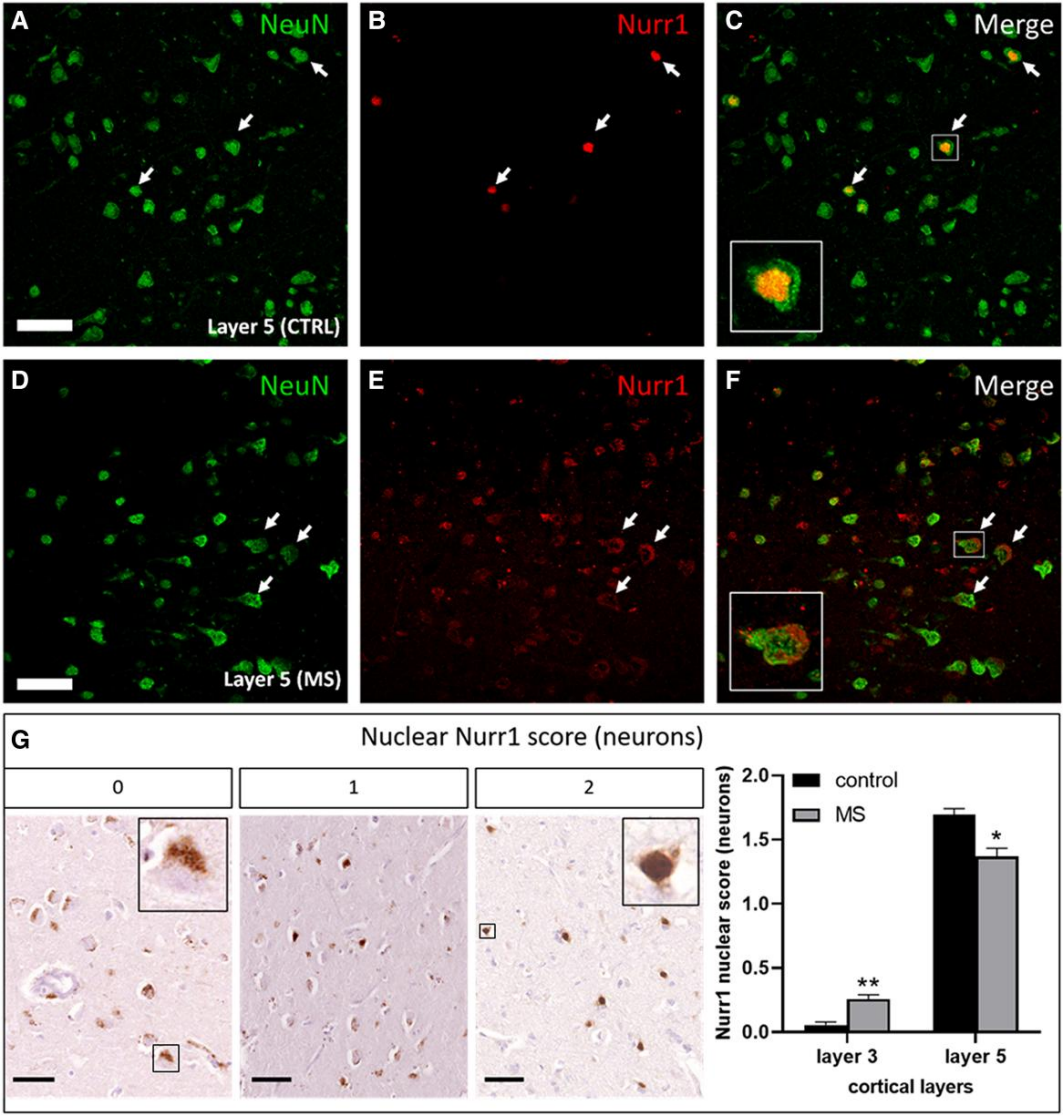
**Representative NeuN+ and Nurr1+ neurons and semi-quantitative scoring on Nurr1 neuronal localisation.** Confocal images of layer 5 pyramidal neurons in (**A**–**C**) control and (**D**–**F**) multiple sclerosis cases are labelled for NeuN (NeuN; **A**, **D**) and Nurr1 (Nurr1; **B**, **E**) at 400 × magnification, and representative merge image (**C**, **F**). Nuclear and cytoplasmic Nurr1 are illustrated in the inserts (arrows and inserts in **C** and **F**, respectively; scale bar 50 µm). (**G**) We devised a semiquantitative score for the localisation of Nurr1 in cytoplasm and nucleus of neurons in layer 3 and layer 5. For each FOV analysed, Nurr1 expression in each compartment was scored on a scale of 0 (mostly cytoplasmic), 1 (cytoplasmic/nuclear) and 2 (mostly nuclear). Cytoplasmic Nurr1 is greater in layer 3 of controls compared with multiple sclerosis cases, while nuclear Nurr1 is greater in layer 5 of controls compared with multiple sclerosis cases (Mann-Whitney tests). Cytoplasmic and nuclear Nurr1 are illustrated in the inserts of score 0 and score 1, respectively. Data presented as mean ± SEM. *P < 0.05; **P < 0.01; scale bar 100 μm.

Nurr1 is expressed in neurons (Fig. 2) and in other non-neuronal brain cells (Supplementary Fig. 3). More specifically, we observed that pyramidal neurons expressed Nurr1 in cortical layers 3 and 5 in both multiple sclerosis and control cases. Importantly, we observed that Nurr1 cytoplasmic expression appears coarser, irregular and widely distributed than lipofuscin (Supplementary Fig. 1). Therefore, it is unlikely that the Nurr1 cytoplasmic expression is solely localised to lipofuscin. Further interrogation revealed differences in the subcellular expression pattern within neurons (i.e. cytoplasmic versus nuclear) (Fig. 2G). Nurr1 was preferentially expressed in neuronal cytoplasm in cortical layer 3 in both multiple sclerosis and control cases while higher Nurr1 nuclear expression in multiple sclerosis (*P* = 0.0068). In contrast, Nurr1 was preferentially expressed in neuronal nuclei in cortical layer 5 in both multiple sclerosis and control cases with Nurr1 cytoplasmic localisation being more common in multiple sclerosis (*P* = 0.045).

No difference in Nurr1 expression was observed in NAWM between multiple sclerosis and control cases (*P* = 0.18). Nurr1 expression did not differ between motor cortical lesional areas and normal-appearing grey matter in multiple sclerosis cases (Supplementary Fig. 2).

### Overall and nuclear Nurr1 expression relates to neuronal survival in multiple sclerosis

**Figure 3:**
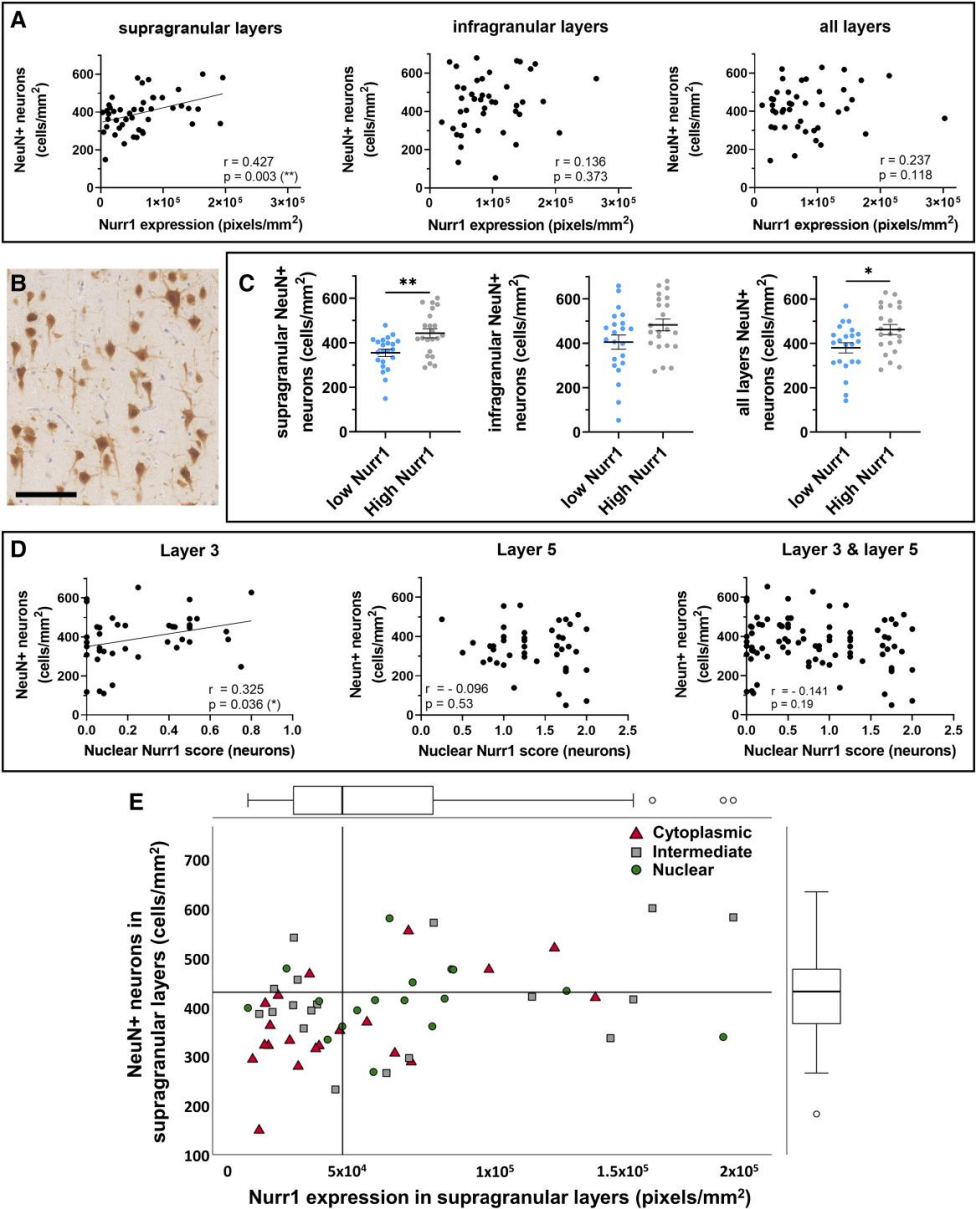
**Relationships between Nurr1 expression and neuronal localisation with neuronal density in multiple sclerosis.** Nurr1 expression is positively correlated with (**A**) NeuN+ neuronal density, which is restricted to supragranular layers in multiple sclerosis (Spearman rank-correlation coefficients). (**B**) Representative labelling of NeuN+ neurons using DAB immunostaining in supragranular layer 3 in multiple sclerosis (scale bar 100 μm). (**C**) Further assessment shows an increase in NeuN+ neuronal density in the multiple sclerosis subgroup with high levels of Nurr1, restricted to supragranular layers (Mann-Whitney tests) (**D**) Nurr1 preferential localisation in nucleus is positively correlated with NeuN+ neuronal densities and restricted to superficial layer 3 (Spearman rank-correlation coefficients). (**E**) Nurr1 expression and sub-cellular localisation interact in predicting NeuN+ neuronal densities, as cases with low NeuN+ neuron densities (below median, horizontal line) tend to have both low Nurr1 expression (below median, vertical line) and a cytoplasmic Nurr1 expression (Generalised estimating equation model). The scatterplot shows the relationship between Nurr1 expression and NeuN+ neuronal density. Dots are classified based on the Nurr1 localisation score as predominant cytoplasmic (higher tertiles), intermediate or nuclear Nurr1 expression (*bottom* tertiles). Distribution of Nurr1 expression (on *top*) and NeuN+ neuronal densities (on the right) are represented with box plots. Data presented as mean ± SEM. *P < 0.05; **P < 0.01; scale bar 100 μm.

Quantitative and semi-quantitative measures of Nurr1 expression were compared with neuronal density throughout NAGM motor cortical layers (Fig. 3).

Despite there being no difference in neuronal densities between multiple sclerosis and controls, as previously published,^12^ we observed differences in the relationships between Nurr1 expression and neuronal density within the multiple sclerosis cohort when considering supra- and infragranular cortical layers (Fig. 3A–C). Nurr1 expression correlated with NeuN+ neurons, a finding restricted to supragranular layers (supragranular layers, *r* = 0.43, *P* = 0.003; infragranular layers, *r* = 0.14, *P* = 0.373; all layers, *r* = 0.24, *P* = 0.118). In fact, when comparing multiple sclerosis cases showing ‘high’ and ‘low’ Nurr1 expression (Fig. 3C), we found that multiple sclerosis cases with low expression showed decreased neuronal densities compared to high expressing cases (*P* = 0.033), restricted to supragranular layers (supragranular layers: *P* = 0.002; infragranular layers: *P* = 0.37). In a subset of multiple sclerosis cases (*n* = 20, Table 1), we found that multiple sclerosis cases with the lowest Nurr1 expression show a decreased neuronal density for GAD+ neurons, also restricted to supragranular layers (Supplementary Fig. 4).

Further, preferential nuclear Nurr1 sub-cellular localisation was correlated with NeuN+ neuronal density in cortical layer 3 (Fig. 3D, layer 3: *r* = 0.32, *P* = 0.036; layer 5: *r* = −0 .10, *P* = 0.53; averaged layer 3–layer 5: *r* = 0.14, *P* = 0.19).

Importantly, Nurr1 expression and Nurr1 nuclear localisation showed an interactive effect in predicting neuronal densities. In a generalised linear model, Nurr1 expression (Wald χ^2^ 13.97, *P* < 0.001), sub-cellular localisation (Wald χ^2^ 6.32, *P* = 0.012) and their interaction (Wald χ^2^ 5.22, *P* = 0.022) predicted NeuN+ neuron density in supragranular layers (LRT χ^2^ 14.39, *P* = 0.002). In fact, the interactive effect of Nurr1 expression and sub-cellular localisation was driven by cases with low neuronal densities, where expression of Nurr1 was low and predominantly cytoplasmic. In contrast, neuronal density is not impacted at higher Nurr1 expression regardless of Nurr1 sub-cellular localisation (Fig. 3E). Importantly, sex, age and post-mortem interval did not contribute to the model nor modify the fit of the model or its predictors. Similar results were obtained when considering layer 3 only (LRT χ^2^ 15.1, *P* = 0.002), while layer 5 only analyses did not reach significance.

### Nurr1 expression is negatively correlated with lymphocytic inflammation in multiple sclerosis

**Figure 4:**
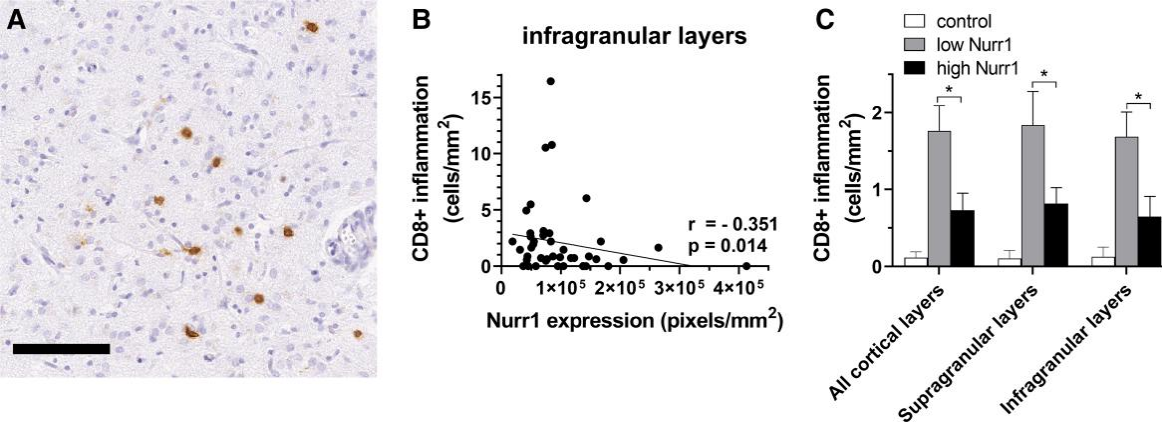
**Relationships between Nurr1 expression and CD8+ lymphocyte density.** (**A**) Representative labelling of CD8+ cells using DAB immunostaining. (**B**) A negative correlation between Nurr1 expression and CD8+ lymphocytes is restricted to infragranular layers in multiple sclerosis cohort (Spearman rank-correlation coefficients). (**C**) Further assessment shows a significant increase in CD8+ lymphocytes in the cases lying at the lowest extreme of Nurr1 expression (*n* = 20) compared with the cases with the highest extreme of Nurr1 expression throughout all cortical layers (Mann-Whitney tests). *P < 0.05; scale bar 100 μm.

As previously reported, CD3+/CD8+ lymphocytic inflammation was increased in multiple sclerosis compared with control cases.^12^ We compared quantitative measures of Nurr1 expression in the extremes of Nurr1 expression (*n* = 20; Table 1) with lymphocytic (CD3+ and CD8+ T-cell) density in all motor cortical layers (Fig. 4).

Nurr1 expression negatively correlated with CD8+ inflammation in multiple sclerosis cases, a finding restricted to infragranular layers (supragranular layers, *r* = −0.110, *P* = 0.472; infragranular layers, *r* = −0.351, *P* = 0.0014; all layers, *r* = −0.371, *P* = 0.012, Fig. 4B). CD8+ inflammation was decreased in cases with highest Nurr1 expression compared to cases with lowest Nurr1 expression in all NAGM layers (supragranular layers, *P* = 0.045; infragranular layers, *P* = 0.029; all layers, *P* = 0.023, Fig. 4C).

In contrast, no correlation between Nurr1 expression and CD3+ inflammation was observed in multiple sclerosis cases or controls (data not shown). No difference in CD3+ inflammation was observed between multiple sclerosis cases displaying high or low Nurr1 expression throughout NAGM layers (supragranular layers, *P* = 0.071; infragranular layers, *P* = 0.057; all layers, *P* = 0.069, data not shown).

### Nurr1 expression is increased in older multiple sclerosis cases, and its nuclear localisation relates to age-at-death and disease severity

Quantitative and semi-quantitative measures of Nurr1 expression in NAGM and their relationship with brain weight, duration of the disease, age-at-death and time to wheelchair can be found in Supplementary Figs. 5 and 6.

In multiple sclerosis, no relationship was observed between Nurr1 expression and brain weight, duration of disease or time to wheelchair. While age-at-death did not correlate with Nurr1 expression when all multiple sclerosis cases were considered, we observed increased Nurr1 expression in cases older than the median age of the cohort (*P* = 0.020).

We found that preferential nuclear localisation of Nurr1 in layer 3 was correlated with age-at-death, duration of the disease and time to wheelchair. Similar findings were observed comparing scores of cytoplasmic and nuclear Nurr1 localisation in layer 3 neurons, while preferential nuclear localisation of Nurr1 in layer 5 only related to increased duration of the disease. No relationship was found between Nurr1 sub-cellular localisation in layer 5 neurons and demographic characteristics (Supplementary Fig. 6).

Multiple sclerosis cases demonstrated decreased brain weight compared to controls as previously published.^13^ Control cases did not show a relationship between Nurr1 expression nor sub-cellular localisation with brain weight or age-at-death.

## Discussion

We introduce motor cortical Nurr1 expression as a potential neuroprotective target in multiple sclerosis pathology. We show that Nurr1 expression is elevated in multiple sclerosis motor cortex and relates to reduced neuronal loss and CD8+ T-cell inflammation in a cortical-layer specific fashion suggesting that the influence of Nurr1 expression in multiple sclerosis is complex and likely involves numerous mechanisms. We also show that increased Nurr1 expression in multiple sclerosis cases with older age-at-death provides support to the notion that Nurr1 may be involved in mechanisms that protect against deleterious inflammation or neurodegeneration. These findings, in conjunction with those in an animal model of multiple sclerosis (experimental autoimmune encephalomyelitis) and others on amyotrophic lateral sclerosis (with some similar features and symptoms) wherein Nurr1 activation reduced severity of disease, provide functional relevance of our findings in human disease.^15,16^ Could enhancing Nurr1 expression in early multiple sclerosis and throughout the disease course limit cytotoxic T-cell inflammation and neuronal loss that contribute to multiple sclerosis progression?

Global Nurr1 expression in the motor cortex in multiple sclerosis and controls was complex. In controls, Nurr1 was predominantly expressed in infragranular layer 5, which is consistent with animal model data that has shown a similar distribution of Nurr1 expression in the brain. In contrast, Nurr1 expression in multiple sclerosis was increased and more widely distributed throughout cortical layers, including more superficial ones, compared to controls. Pathological studies have consistently shown that meningeal inflammation is a common feature with some suggesting that cytotoxic factors diffuse from the meninges into superficial cortical layers and contribute to neuronal loss.^17,18^ Further, work from our group and others has shown that surrogate markers of blood–brain barrier disruption (i.e. fibrinogen) are most extensively deposited in deeper cortical layers and associated with neuronal loss.^13^ The upregulation of Nurr1 expression in both superficial and deep cortical layers could be a response to these deleterious processes that impact neuronal health and survival.^19,20^ Given the established anti-inflammatory role of Nurr1 in the brain, the heightened expression of this protein throughout the multiple sclerosis motor cortex supports a potential neuroprotective role. These findings also point to Nurr1 expression being controlled, at least in part, by layer-specific factors, which adds further complexity to these findings. These important differences in the expression pattern go beyond the resolution of *in vivo* technologies, which may explain the conflicting reports about Nurr1 expression in blood samples from people with multiple sclerosis compared with controls.^21,22^

Nurr1 appears to relate to neuropathological features in multiple sclerosis. The expression of Nurr1 within our multiple sclerosis cohort was related to NeuN+ neuronal density and the GABAergic (GAD+) neuronal subpopulation, suggesting a neuroprotective role. This finding is in line with previous studies that show that Nurr1 exerts a protective effect on GABAergic neurons *in vitro*^6^ and facilitates maintenance of dopaminergic neurons, a GABAergic subtype.^23^ In addition, the homogeneous distribution of Nurr1 in functionally-relevant layer 5 and its expression in projection neurons, in both multiple sclerosis and controls, supports that Nurr1 may play a role in preserving motor function.^24^ This is reinforced by on-going clinical trials highlighting the contribution of Nurr1 on neuronal survival in Parkinson’s disease,^25^ and previous work on experimental autoimmune encephalomyelitis mouse models, where enhanced Nurr1 expression delayed the onset and reduced the severity of motor disability by reducing neuronal loss.^15^ Interestingly, no differences in Nurr1 expression were found in lesional areas compared to NAGM in multiple sclerosis suggesting that factors other than demyelination are pertinent to Nurr1’s relationship to neuronal survival.

An intriguing finding of this study is the observed differences in the impact of Nurr1 depending on its cortical layer/neuronal sub-cellular localisation in multiple sclerosis cortex. We found that Nurr1 expression and its nuclear localisation relate to neuronal survival in supragranular layers, while Nurr1 expression negatively relates to CD8+ inflammation throughout all cortical layers, particularly in infragranular layers. Cortical layer-specific differences in Nurr1 expression and its relationship to inflammation and neurodegeneration suggest that local tissue factors influence the relationship between Nurr1 and inflammatory and neurodegenerative mechanisms in multiple sclerosis. Indeed, our findings show an inverse pattern of Nurr1 cytoplasmic or nuclear sub-cellular localisation in pyramidal neurons in superficial layer 3 and deep layer 5. As Nurr1 is a transcription factor mainly playing its role in the nucleus, we hypothesise that the shuttling from nuclear to cytoplasmic localisation of Nurr1 in layer 5 neurons may have an impact in motor function in multiple sclerosis. Conversely, the enhanced nuclear expression of Nurr1 in cortical layer 3 may serve to protect neurons from the inflammatory insults known to impact superficial cortical layers. A protective role of Nurr1 is further supported by our data linking heightened Nurr1 expression with older age-at-death, and the relationship between nuclear localisation of Nurr1 and older age-at-death, longer disease duration and longer time-to-wheelchair in our multiple sclerosis cohort. Importantly, our data also show that interaction of Nurr1 expression with its nuclear localisation relates to increased neuronal survival. Functional validation of such a protective role of nuclear Nurr1 expression is corroborated in animal models of amyotrophic lateral sclerosis where nuclear Nurr1 translocation occurred in early disease stages where spinal motor neuronal loss was not severe.^15^ The conflation of these findings suggests that enhancing Nurr1 expression in the nuclear compartment of motor neurons may provide a putative protective mechanism to halt neurodegeneration and therefore, disability progression in multiple sclerosis.

Considering that neurodegeneration is closely linked to inflammation in multiple sclerosis, our finding that Nurr1 expression negatively relates to cytotoxic CD8+ inflammation in multiple sclerosis cortex provides support to the possibility that Nurr1’s putative neuroprotective role may be mediated, at least in part, by its influence on T-cell inflammation. In line with this, it has been shown that Nurr1 upregulates Foxp3 in Treg cells,^26^ a T-cell subset that is known to suppress CD8+ T cell function, differentiation and expansion.^27^ Altogether, this suggests that Nurr1 may exert some of its neuroprotective function by modulating the CD8+ T lymphocyte subpopulation in multiple sclerosis cortex.

In summary, motor cortical Nurr1 expression is elevated in multiple sclerosis motor cortex and relates to reduced neuronal loss and CD8+ T-cell inflammation in a cortical-layer specific fashion suggesting a possible neuroprotective role that likely involves numerous mechanisms.^21^ Future work should be aimed to better understand the mechanisms that alter Nurr1 expression at different stages and forms of multiple sclerosis pathology, such as relapsing–remitting multiple sclerosis and primary progressive multiple sclerosis. Further, better characterisation of its provenance, its differential cellular expression (astrocytes, microglia, oligodendrocytes and neurons) and factors impacting its sub-cellular localisation will cast light on the role of Nurr1 as a potential neuroprotective target against inflammation and neurodegeneration to halt the often-devastating consequences of the disease.

## Supplementary Material

fcad072_Supplementary_Data

## Data Availability

The data that support the findings of this study are available from the corresponding author upon reasonable request.

## Abbreviations

DAB =: 3,3′-diaminobenzidine
FFPE =: formalin-fixed paraffin-embedded
FOV =: field of view
IF =: immunofluorescence
NAGM =: normal-appearing grey matter
NAWM =: normal-appearing white matter
Nurr1 =: nuclear receptor-related factor 1
PM =: post-mortem
SEM =: standard error of the mean
