# Supplementary material for: A potential protective role of the nuclear receptor-related factor 1 (Nurr1) in multiple sclerosis motor cortex: a neuropathological study
Source: Brain Commun. 2023 Mar 17;5(2):fcad072. doi: 10.1093/braincomms/fcad072 (PMC10088483; doi:10.1093/braincomms/fcad072)
Supplement: fcad072_Supplementary_Data [file fcad072_supplementary_data.pdf]

Supplementary material for:

A potential protective role of the nuclear receptor-related factor 1 (Nurr1) in multiple sclerosis motor cortex: a neuropathological study

Jonathan Pansieri[1], Marco Pisa[1], Richard L. Yates[1], Margaret M. Esiri[1], Gabriele C. DeLuca[1]

[1] Nuffield Department of Clinical Neurosciences, University of Oxford, Level 6 West Wing, 6 John Radcliffe Hospital, Oxford, UK

Correspondence to: Gabriele C. De Luca, MD, DPhil, FRCPath, FAAN

Professor of Clinical Neurology and Experimental Neuropathology, Nuffield Department of Clinical Neurosciences, University of Oxford. Director, Clinical Neurosciences Undergraduate Education, Oxford Medical School, Level 6 West Wing, John Radcliffe Hospital, Oxford, UK OX3 9DU, UK

E-mail: gabriele.deluca@ndcn.ox.ac.uk

Supplementary Figures

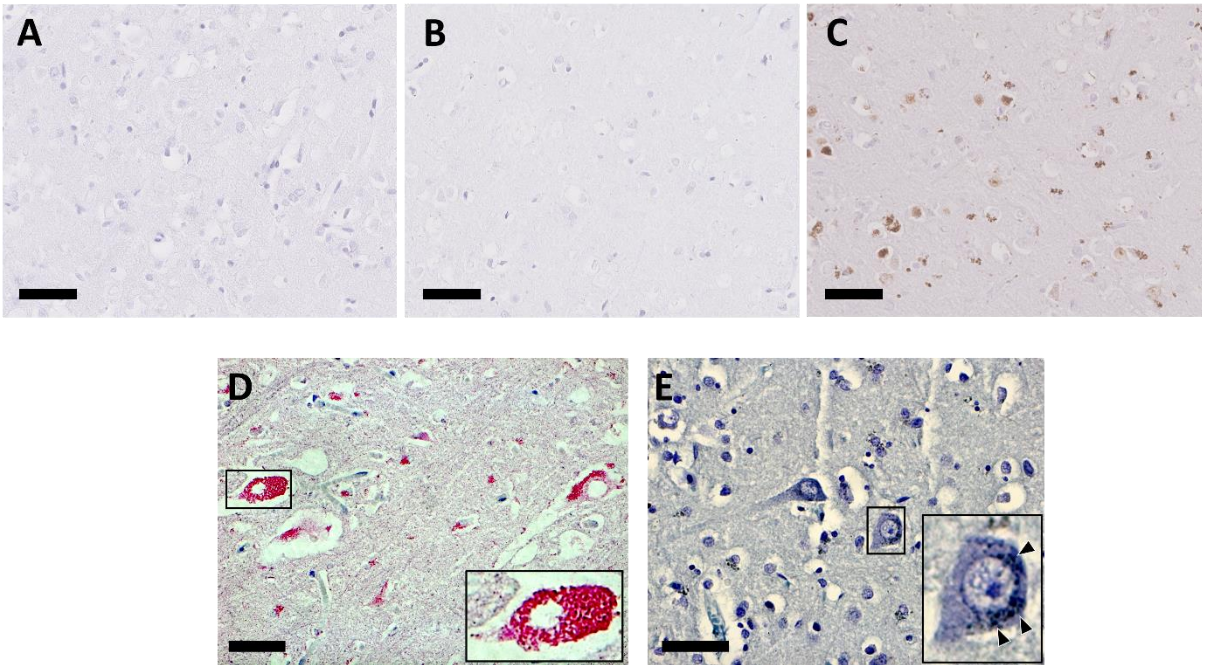

Supplementary Fig.1 Nurr1 cytoplasmic expression in neurons of cortical layer 5 from MS case. (A-C) Representative immunolabelling of Nurr1 revealed by DAB with omission of **(A)** primary or **(B)** secondary antibodies, showing no staining. **(C)** Representative immunolabelling of Nurr1 revealed by DAB staining (brown). **(D)** Representative immunolabelling of Nurr1 revealed by Vector red staining (purple) shows widely distributed, irregular and coarse granules within cytoplasm of neurons compared with **(E)** lipofuscin staining revealed by Sudan Black B staining (black, arrowheads in the insert). Scale bars 50 µm.

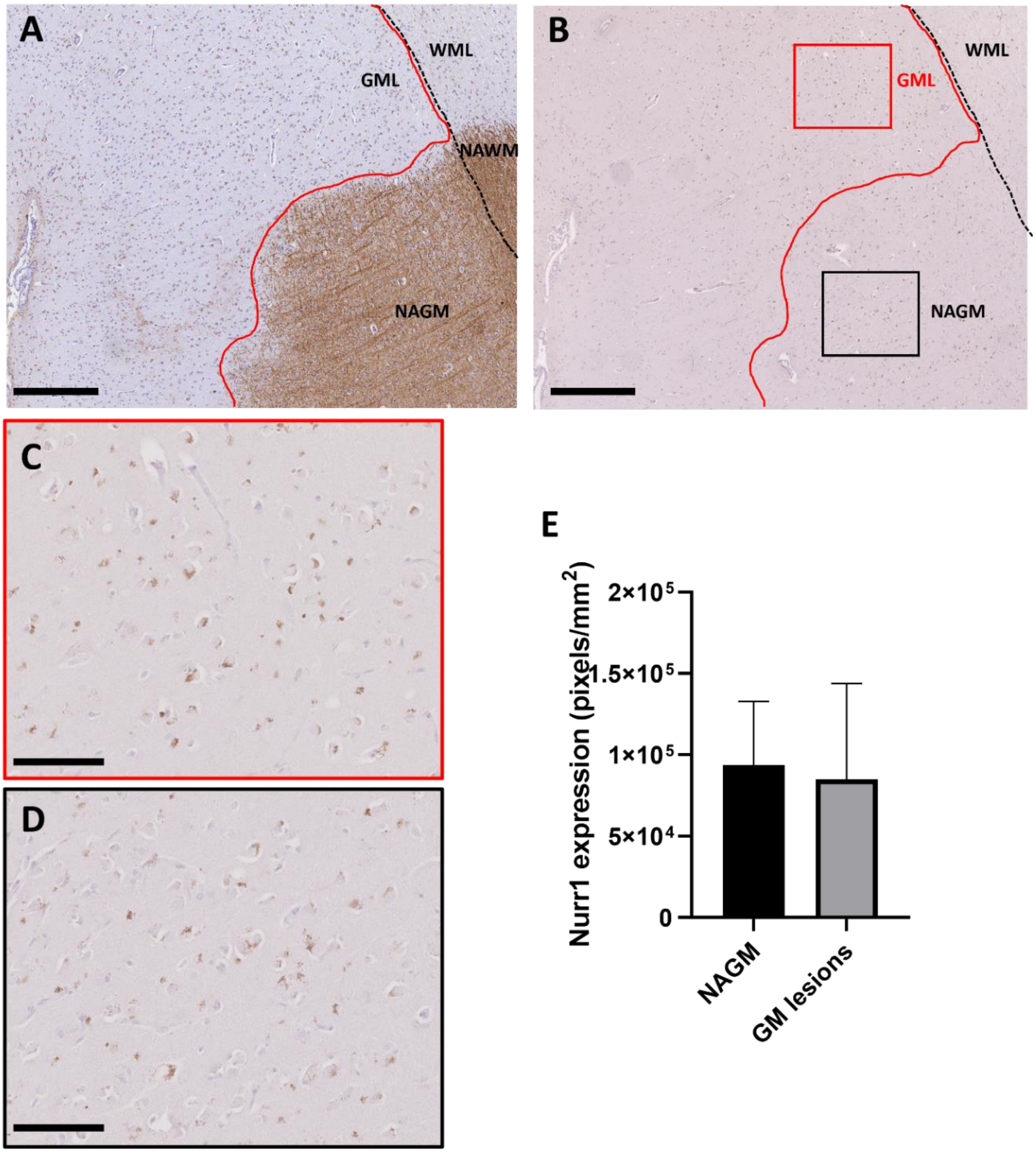

Supplementary Fig.2 No difference of Nurr1 expression comparing NAGM with GM lesions (A-D) Representative immunolabelling of **(A)** PLP and **(B)** Nurr1 revealed by DAB illustrating the demyelinated areas (red line) and corresponding Nurr1 expression, respectively (Scale bar 400µm). **(C,D)** Higher magnification of Nurr1 expression in **(C)** GM lesions and **(D)** NAGM, illustrating the absence of difference in Nurr1 expression in NAGM and GM lesions (Scale bar 100µm). **(E)** No differences in Nurr1 expression comparing NAGM and GM lesions was found in MS cases showing large demyelinated areas (Wilcoxon test, n=18, p = 0.09). (NAWM = normal-appearing white matter ; NAGM = normal-appearing grey matter ; WML = white matter lesion ; GML = grey matter lesion ; border between WM and GM is delineated by dotted line).

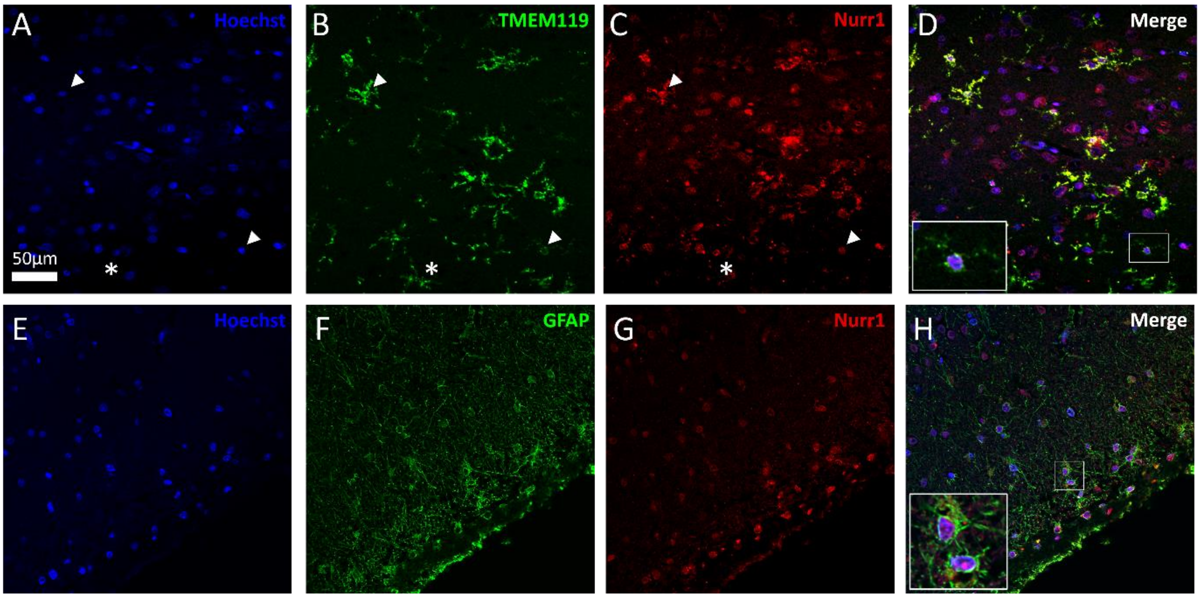

Supplementary Fig. 3. Co-localisation of Nurr1 with glial cells. (A-D) Confocal images of layer 3 microglia in MS case are labelled for nucleus (Hoechst, blue, **A**), microglia (TMEM119, green, **B**), and Nurr1 (Nurr1, red, **C**) at 400x magnification, and representative merge image **(D).** Arrowheads indicates co-labelling of Nurr1 with TMEM119+ microglia, while asterisk illustrate TMEM119+ microglia without Nurr1 co-localisation. Intra-cellular Nurr1 is illustrated in the insert. **(E-F)** Confocal images of layer 1-2 astrocytes in MS case are labelled for nucleus (Hoechst, blue, **E**), astrocytes (GFAP, green, **F**), and Nurr1 (Nurr1, red, **G**) at 400x magnification, and representative merge image **(H).** Nuclear and cytoplasmic Nurr1 is illustrated in the insert.

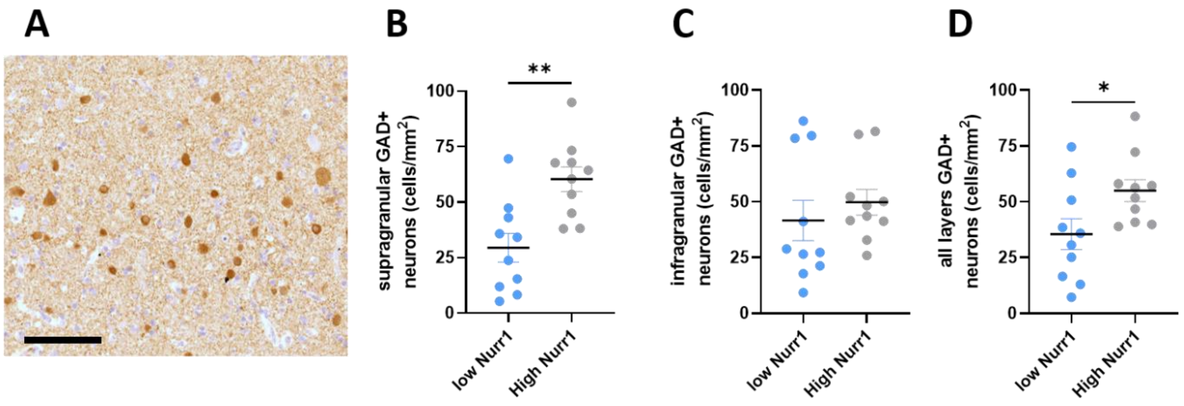

Supplementary Fig. 4. Relationships between Nurr1 expression and GAD+ neuronal density in MS cases lying at the lowest extremes of Nurr1 expression. (A) Representative labelling of GAD+ neurons using DAB immunostaining in supragranular layer 3 in MS. **(B-D)** Further assessment shows increased density of GAD+ neurons in the MS subgroup with high levels of Nurr1, a finding restricted to supragranular layers. (Mann-Whitney test, data presented as mean ± SEM.* $p < 0.05$, **$p < 0.01$; Scale bar 100 µm).

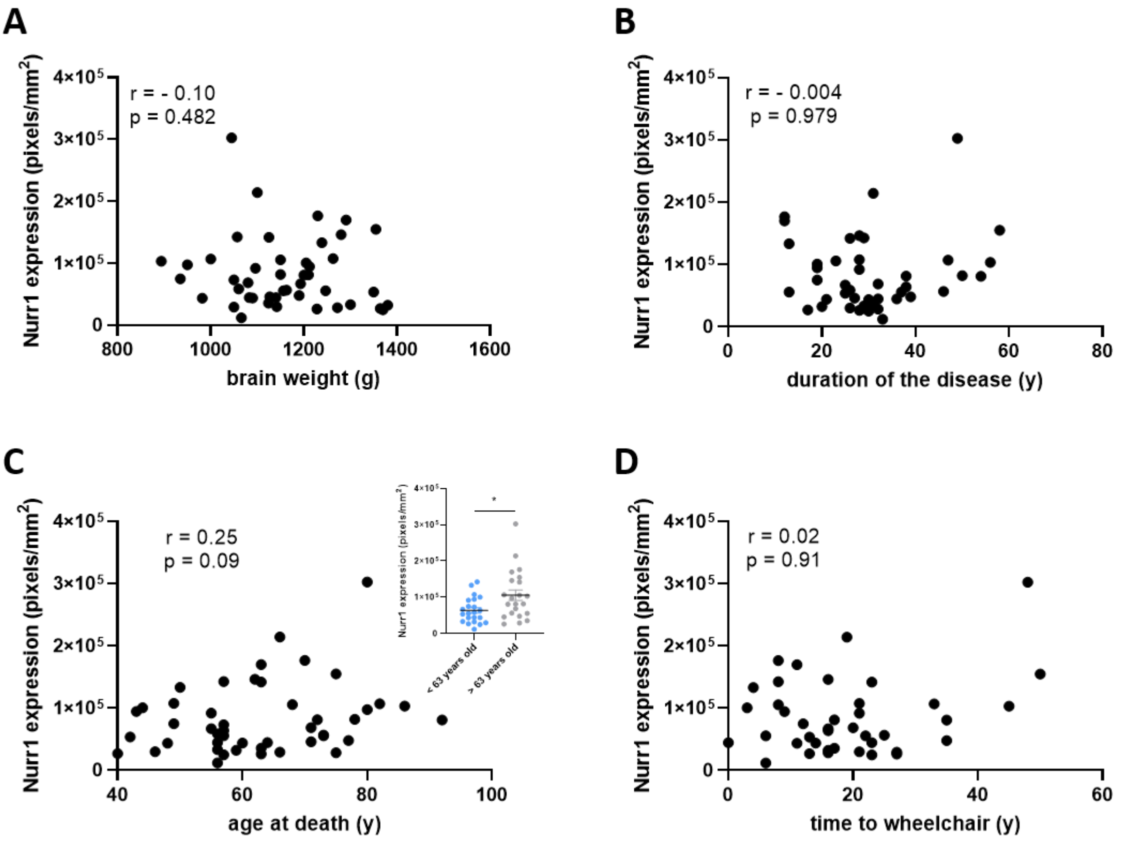

Supplementary Fig. 5. Relationship of Nurr1 expression with clinical features in MS. No correlation was found between Nurr1 expression and **(A)** brain weight, **(B)** duration of the disease, **(C)** age at death and **(D)** time to wheelchair. However, Nurr1 expression in NAGM is greater in cases older than median age (insert in **C,** Mann-Whitney test, $p = 0.019$). (Spearman rank-correlation coefficient was used in A-D ; * $p < 0.05$)

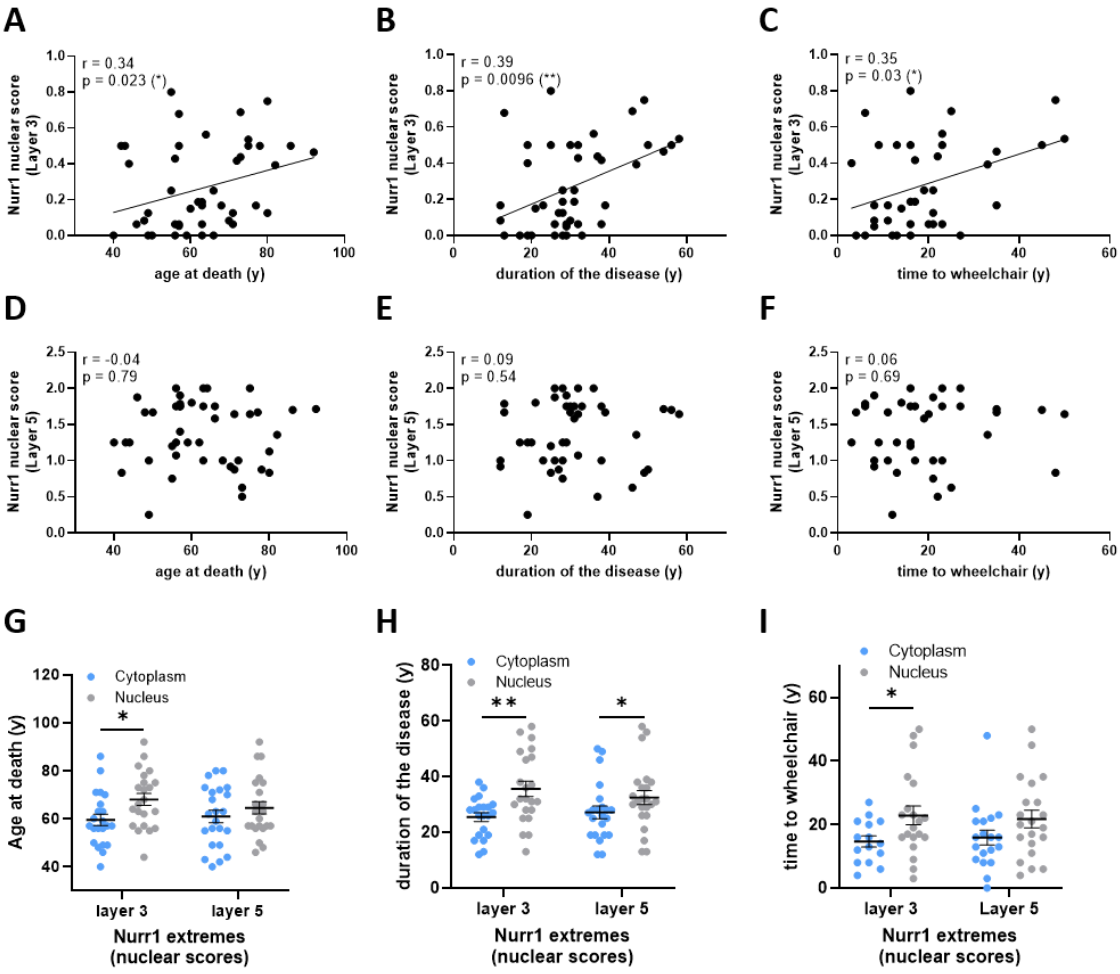

Supplementary Fig. 6. Relationship of Nurr1 neuronal localisation with clinical features in MS. (A-C) Correlation was found between Nurr1 nuclear score in layer 3 and age at death, duration of the disease and time to wheelchair (Spearman rank-correlation coefficients), while **(D-F)** no correlation was found between Nurr1 nuclear score in layer 5 and these features (Spearman rank-correlation coefficients). **(G)** Only Nurr1 preferential nuclear location in layer 3 show increased age at death (Mann-Whitney test ; layer 3 : p = 0.019 ; layer 5 : p = 0.36), while **(H)** both preferential Nurr1 nuclear location in layer 3 and layer 5 show increased duration of the disease (Mann-Whitney test ; layer 3: p = 0.006 ; layer 5 : p = 0.03), and **(I)** only Nurr1 nuclear location in layer 3 show increased time to wheelchair (Mann-Whitney test ; layer 3: p = 0.044 ; layer 5 : p = 0.12). (* p < 0.05, ** p < 0.01)
